# Point prevalence survey to evaluate the seropositivity for coronavirus disease 2019 (COVID-19) among high-risk healthcare workers

**DOI:** 10.1017/ice.2020.1370

**Published:** 2020-12-15

**Authors:** Norihiro Yogo, Kristina L. Greenwood, Leslie Thompson, Pamela J. Wells, Stephen Munday, Tyler C. Smith, Besa Smith, Omid R. Bakhtar

**Affiliations:** 1 Division of Infectious Disease, Sharp Rees-Stealy, San Diego, California; 2 Sharp HealthCare, San Diego, California; 3 Outcomes Research Institute, Sharp HealthCare, San Diego, California; 4 Employee Occupational Health Department, Sharp HealthCare, San Diego, California; 5 Chief Nursing Officer and Vice President, Sharp Memorial Hospital, San Diego, California; 6 Division of Occupational Health, Sharp Rees-Stealy, San Diego, California; 7 Analydata, San Diego, California; 8 Outreach Laboratory Services, Sharp Healthcare, San Diego, California; 9 Pacific Rim Pathology, San Diego, California

## Abstract

Among 1,770 healthcare workers serving in high-risk care areas for coronavirus disease 2019 (COVID-19), 39 (2.2%) were seropositive. Exposure to severe acute respiratory coronavirus virus 2 (SARS-CoV-2) in the community was associated with being seropositive. Job or unit type and percentage of time working with COVID-19 patients were not associated with positive antibody tests.

Currently, the mainstay of diagnosis for severe acute respiratory syndrome coronavirus 2 (SARS-CoV-2) causing coronavirus disease 2019 (COVID-19) is polymerase chain reaction (PCR) of respiratory tract samples. However, due to limited testing at the onset of the epidemic in the US and asymptomatic courses of infection,^1^ an accurate accounting of all individuals exposed to SARS-CoV-2 is an area of ongoing investigation. Large seroprevalence surveys have been conducted to better assess epidemiologic exposure to SARS-CoV-2.^2^ However, these studies have focused on the general population and have not been specific to healthcare workers (HCWs), who accounted for ~11% of cases early in the pandemic.^3^ Identifying seroprevalence among HCWs may provide insights into exposure to SARS-CoV-2 and effectiveness of infection control policies.

## Methods

### Study setting

Sharp HealthCare is a multidisciplinary healthcare system in San Diego County, with 4 acute-care hospitals, an inpatient behavioral health hospital, and 3 skilled nursing facilities. Over the study period, the average system-wide daily census of COVID-19 positive patients was 87: 43% at Chula Vista (southern San Diego), 27% each at Grossmont (eastern) and Metropolitan (central), and 3% at Coronado (bay area).

This point prevalence study occurred from May 20 through June 8, 2020. Institutional review board review was obtained prior to study enrollment.

### Infection control measures

All COVID-19 confirmed cases and persons under investigation were placed in negative pressure rooms with airborne and contact precautions. Visitors were excluded from our hospitals starting March 18, 2020. Telemedicine was made available starting March 19, 2020. Permissive masking for HCWs began on March 30, 2020. Universal masking for all patients and staff, regardless of COVID-19 status, began April 22, 2020.

### Inclusion and exclusion criteria

HCWs with direct contact to patients with COVID-19 and those working in congregate care areas were invited to this study. High-risk care occupational areas were defined as intensive care units, COVID-19 designated acute-care units, and emergency departments. Congregate care areas were defined as nursing facilities and behavioral health units. Additionally, respiratory therapists, anesthesiologists, and endoscopy technicians at highest exposure to aerosol-generating procedures were included. Phlebotomists were also included given the large volume of direct patient exposure, including COVID-19–related care areas.

Staff without direct patient care responsibilities and HCWs with active symptoms of COVID-19 were excluded from the study.

### Study design

Study participants were invited through the hospitals’ Employee Occupational Health Department. Study participants were instructed on how to perform a self-collected nares PCR-based test and collection was supervised by a study nurse. A paired nurse-drawn serum for antibody testing was also collected. Study participants were asked to complete a study questionnaire to report demographic information and prior COVID-19 testing or exposures. If study participants did not report working in prespecified high-risk care areas, survey data were cross referenced with the EOHD database to ensure accuracy.

### Laboratory methods

Nasal PCRs were conducted using Roche SARS CoV-2 qualitative real-time PCR (Cobas 6800 platform, Roche Diagnostics, Indianapolis, IN). Serology testing was performed using Roche Elecsys Anti-SARS-CoV-2 (Cobas platform) immunoassay. The immunoassay utilizes “high-affinity” antibodies, with a reported specificity of 99.8% and sensitivity of 100% at >14 days after PCR confirmation.

### Statistical analysis

Unadjusted associations between all characteristics and the outcomes of a positive PCR test or a positive antibody test were explored using the Pearson χ^2^ or the Fisher exact test, with *P* < .05 level of statistical significance, using SAS version 9.4 software (SAS Institute, Cary, NC). Additional univariate and bivariate analyses compared participant characteristics and outcomes based on previous history of COVID-19. Saturated multivariable logistic regression models were used to investigate the adjusted odds of positive PCR or antibody tests. C-statistics were used to measure the model’s discriminatory value.

## Results

Overall, 4,258 HCWs were invited to this study, of whom 1,897 participated. However, 127 were excluded: 75 were non–bedside-care staff, 47 did not work in high-risk care areas, and 5 had incomplete survey results. None of the excluded participants had a positive antibody or PCR result.

Demographic and survey results of the 1,770 study participants included are described in Table 1. In total, 39 study participants (2.2%) had a positive antibody test. Among 22 participants who reported a history of presumptive or confirmed COVID-19, 14 were antibody positive; none had a positive PCR test. Of 1,748 study participants without a reported history of COVID-19, 23 had a positive antibody test only (1.3%), 2 had a positive PCR test only (0.1%), and 2 had both a positive PCR and antibody test (0.1%). All 4 cases of positive PCR tests were from Chula Vista, which coincided with a cluster of cases among HCWs.

**Table 1.** Demographics and Clinical Characteristics of Survey Participants by Positive Antibody Test and Positive PCR Test for COVID19 Among 1,770 Healthcare Providers

Characteristic	Overall (n=1,770), No. (%)	Antibody Result, No. (%)		PCR Result, No. (%)	
		Negative (n=1,731)	Positive (n=39)	Negative (n=1,766)	Positive (n=4)
**Sex**					
Female	1,337 (75.5)	1,309 (75.6)	28 (71.8)	1,335 (75.6)	2 (50.0)
Male	433 (24.5)	422 (24.4)	11 (28.2)	431 (24.4)	2 (50.0)
**Age group, y**					
18–35	802 (45.3)	784 (45.3)	18 (46.2)	799 (45.2)	3 (75.0)
36–45	460 (26.0)	452 (26.1)	8 (20.5)	460 (26.1)	0 (0.0)
45–55	323 (18.3)	314 (18.1)	9 (23.1)	322 (18.2)	1 (25.0)
56–65	146 (8.2)	144 (8.3)	2 (5.1)	146 (8.3)	0 (0.0)
>65	39 (2.2)	37 (2.1)	2 (5.1)	39 (2.2)	0 (0.0)
**Race/Ethnicity**					
African American/Black	58 (3.3)	55 (3.2)	3 (7.7)	58 (3.3)	0 (0.0)
Asian	510 (28.8)	500 (28.9)	10 (25.6)	510 (28.9)	0 (0.0)
Caucasian	721 (40.7)	709 (41.0)	12 (30.8)	721 (40.8)	0 (0.0)
Hispanic/Latino	317 (17.9)	305 (17.6)	12 (30.8)	314 (17.8)	3 (75.0)
Native American	6 (0.3)	6 (0.4)	0 (0.0)	6 (0.3)	0 (0.0)
Pacific Islander	73 (4.1)	73 (4.2)	0 (0.0)	73 (4.1)	0 (0.0)
Other	80 (4.5)	78 (4.5)	2 (5.1)	79 (4.5)	1 (25.0)
Decline to state	5 (0.3)	5 (0.3)	0 (0.0)	5 (0.3)	0 (0.0)
**Previous diagnosis of COVID-19**					
No	1,748 (98.8)	**1,723** (99.5)	**25 (64.1)**	1,744 (98.8)	4 (100.0)
Yes	22 (1.2)	**8** (0.5)	**14 (35.9)**	22 (1.2)	0 (0.0)
**Previous PCR test for SARS-CoV-2**					
No	1,598 (90.3)	**1,574** (90.9)	**24 (61.5)**	1,594 (90.3)	4 (100.0)
Yes	172 (9.7)	**157** (9.1)	**15 (38.5)**	172 (9.7)	0 (0.0)
Previous positive PCR result	15 (8.7)	2 (1.3)	13 (86.7)	15 (100.0)	0(0.0)
Previous negative PCR result	157 (91.3)	155 (98.7)	2 (13.3)	157 (100.0)	0 (0.0)
**Previous antibody test for SARS-CoV-2**					
No	1,736 (98.1)	1,697 (98.0)	39 (100.0)	1,732 (98.1)	4 (100.0)
Yes	34 (1.9)	34 (2.0)	0 (0.0)	34 (1.9)	0 (0.0)
Previous antibody test positive	1 (2.9)	1 (2.9)	0 (0.0)	1 (2.9)	0 (0.0)
Previous antibody test negative	33 (97.1)	33 (97.1)	0 (0.0)	33 (97.1)	0 (0.0)
**Known community exposure**					
No	1,710 (96.6)	**1,676** (96.8)	**34 (87.2)**	1,706 (96.6)	4 (100.0)
Yes	60 (3.4)	**55** (3.2)	**5 (12.8)**	60 (3.4)	0 (0.0)
**% working with COVID-19 patients**					
0%	124 (7.0)	121 (7.0)	3 (7.7)	124 (7.0)	0 (0)
1%–25%	287 (16.2)	280 (16.2)	7 (17.9)	287 (16.3)	0 (0)
26%–50%	212 (12.0)	207 (12.0)	5 (12.8)	212 (12.0)	0 (0.0)
51%–75%	278 (15.7)	275 (15.9)	3 (7.7)	277 (15.7)	1 (25.0)
76%–100%	869 (49.1)	848 (49.0)	21 (53.8)	866 (49.0)	3 (75.0)
**Exposed without PPE**					
No	1,544 (87.2)	1,512 (87.3)	32 (82.1)	1,540 (87.2)	4 (100.0)
Yes	226 (12.8)	219 (12.7)	7 (17.9)	266 (12.8)	0 (0.0)
**Primary facility**					
Skilled nursing facilities	27 (1.5)	27 (1.6)	0 (0.0)	27 (1.5)	0 (0.0)
Chula Vista inpatient hospital	460 (26.0)	445 (25.7)	15 (38.5)	456 (25.8)	4 (100.0)
Coronado inpatient hospital	90 (5.1)	88 (5.1)	2 (5.1)	90 (5.1)	0 (0.0)
Grossmont inpatient hospital	521 (29.4)	515 (29.8)	6 (15.4)	521 (29.5)	0 (0.0)
Central/Metropolitan inpatient hospital	516 (29.2)	506 (29.2)	10 (25.6)	516 (29.2)	0 (0.0)
Inpatient behavioral health hospital	156 (8.8)	150 (8.7)	6 (15.4)	156 (8.8)	0 (0.0)
**Job category**					
Nurse	1,129 (63.8)	1,101 (63.6)	28 (71.8)	1,127 (63.8)	2 (50.0)
Nurse practitioner	12 (0.7)	12 (0.7)	0 (0.0)	12 (0.7)	0 (0.0)
Nursing assistant	154 (8.7)	149 (8.6)	5 (12.8)	153 (8.7)	1 (25.0)
Phlebotomist	106 (6.0)	106 (6.1)	0 (0.0)	106 (6.0)	0 (0.0)
Physician	110 (6.2)	108 (6.2)	2 (5.1)	110 (6.2)	0 (0.0)
Respiratory therapist	121 (6.8)	121 (7.0)	0 (0.0)	121 (6.9)	0 (0.0)
Social worker, case manager, office staff	35 (2.0)	35 (2.0)	0 (0.0)	35 (2.0)	0 (0.0)
Technician	65 (3.7)	62 (3.6)	3 (7.7)	64 (3.6)	1 (25.0)
Therapist	22 (1.2)	21 (1.2)	1 (2.6)	22 (1.2)	0 (0.0)
Other	16 (0.9)	16 (0.9)	0 (0.0)	16 (0.9)	0 (0.0)
**Primary unit**					
High-risk direct COVID care units	1,304 (73.7)	1,273 (73.5)	31 (79.5)	1,300 (73.6)	4 (100.0)
Emergency department	439 (24.8)	431 (24.9)	8 (20.5)	438 (24.8)	1 (25.0)
Medical COVID unit	495 (28.0)	479 (27.7)	16 (41.0)	492 (27.9)	3 (75.0)
Medical intensive care unit	262 (14.8)	258 (14.9)	4 (10.3)	262 (14.8)	0 (0.0)
Surgical intensive care unit	108 (6.1)	105 (6.1)	3 (7.7)	108 (6.1)	0 (0.0)
Behavioral health	188 (10.6)	181 (10.5)	7 (17.9)	188 (10.6)	0 (0.0)
Endoscopy	38 (2.1)	37 (2.1)	1 (2.6)	38 (2.2)	0 (0.0)
Laboratory, microbiology or pathology	100 (5.6)	100 (5.8)	0 (0.0)	100 (5.7)	0 (0.0)
Skilled nursing facility	63 (3.6)	63 (3.6)	0 (0.0)	63 (3.6)	0 (0.0)
Other	77 (4.4)	77 (4.4)	0 (0.0)	77 (4.4)	0 (0.0)

Note. PPE, personal protective equipment. Bolded numbers indicate statistical significance was found based on *P* < .05 Pearson χ^2^ or Fisher exact tests.

In multivariate analysis, a known community exposure to COVID-19 and Hispanic/Latino participants were associated with seropositivity (Table 2). Percentage of time working with COVID-19 patients, unintentional exposure to COVID-19 without PPE, geographic location, job type and unit were not associated with increased odds of being antibody or PCR positive.

**Table 2.** Multivariable Logistic Regression for Adjusted Odds of PCR or Ab Positive in 1,770 Healthcare Providers and 1,748 Healthcare Providers with No Previous Diagnosis of COVID-19

Characteristic	Odds of Positive PCR or Ab			
	All study participants (No. positive, 41; C-statistic, 0.86)		No previous diagnosis of COVID-19 (No. positive, 27; C-statistic, 0.80)	
	No.	OR (95% CI)	No.	OR (95% CI)
**Sex**				
Female	29	1.00	21	1.00
Male	12	1.00 (0.40–2.51)	6	0.91 (0.33–2.52)
**Age group, y**				
18–35	20	1.00	18	1.00
36–45	8	0.47 (0.17–1.33)	4	0.39 (0.12–1.25)
45–55	9	0.50 (0.16–1.59)	3	0.45 (0.12–1.64)
56–65	2	0.32 (0.04–2.50)	0	…
>65	2	1.93 (0.25–14.80)	2	4.41 (0.79–24.74)
**Race/Ethnicity**				
African American/Black	3	1.32 (0.15–11.47)	1	2.41 (0.27–21.59)
Asian	10	1.16 (0.41–3.31)	7	1.38 (0.46–4.12)
Caucasian	12	1.00	8	1.00
Hispanic/Latino	13	2.79 (1.02–7.65)	9	3.31 (1.11–9.92)
Native American	0	…	0	…
Pacific Islander	0	…	0	…
Other	3	2.85 (0.62–13.15)	2	3.29 (0.64–16.91)
Decline to state	0	…	0	…
**Previous diagnosis**				
No	27	1.00		
Yes	14	117.01 (28.02–488.59)		
**Previous PCR test for SARS-CoV-2**				
No	26	1.00	25	1.00
Yes	15	1.90 (0.67–5.41)	2	0.90 (0.20–4.00)
**Previous antibody test for SARS-CoV-2**				
No	41	1.00	27	1.00
Yes	0	…	0	…
**Known community exposure**				
No	36	1.00	24	1.00
Yes	5	4.41 (1.23–15.82)	3	4.94 (1.27–19.21)
**% working with COVID-19 patients**				
0%	3	1.00	1	1.00
1–25%	7	1.14 (0.16–8.14)	3	1.14 (0.09–14.95)
26–50%	5	1.62 (0.15–18.07)	3	2.60 (0.14–50.03)
51–75%	4	1.14 (0.10–13.72)	4	2.53 (0.13–49.36)
76–100%	22	1.49 (0.14–15.71)	16	2.77 (0.15–50.74)
**Exposed without PPE**				
No	34	1.00	23	1.00
Yes	7	1.17 (0.42–3.26)	4	0.95 (0.30–2.96)
**Primary location**				
Skilled nursing facilities	0	…	0	…
Chula Vista inpatient hospital	17	1.00	11	1.00
Coronado inpatient hospital	2	0.80 (0.12–5.42)	1	0.62 (0.07–5.77)
Grossmont inpatient hospital	6	0.49 (0.15–1.63)	5	0.56 (0.17–1.86)
Central/Metropolitan inpatient hospital	10	0.90 (0.33–2.44)	8	0.90 (0.33–2.49)
Inpatient behavioral health hospital	6	0.14 (0.01–1.87)	2	0.11 (0.01–1.88)
**Job category**				
Nurse	29	1.00	22	1.00
Nurse practitioner	0	…	0	…
Nursing assistant	5	0.65 (0.17–2.45)	2	0.52 (0.12–2.38)
Phlebotomist	0	…	0	…
Physician	2	1.19 (0.19–7.38)	1	1.01 (0.10–9.88)
Respiratory therapist	0	…	0	…
Social worker, case manager, office staff	0	…	0	…
Technician	4	1.65 (0.38–7.15)	2	1.22 (0.22–6.83)
Therapist	1	0.78 (0.01–70.94)	0	…
Other	0	…	0	…
**Primary unit**				
High-risk COVID-19 care units	33	1.00	23	1.00
Behavioral health	7	7.37 (0.55–98.76)	3	10.05 (0.62–161.84)
Endoscopy	1	1.46 (0.13–16.25)	1	2.39 (0.19–29.60)
Laboratory, microbiology or pathology	0	…	0	…
Skilled nursing facility	0	…	0	…
Other	0	…	0	…

Note. PPE, personal protective equipment. OR, odds ratio; CI, confidence interval. High-risk COVID care units defined as emergency department, intensive care units and medical COVID units. ORs and 95% CIs were adjusted for all other variables in the model.

## Discussion

Among high-risk HCWs, we found an antibody positive rate of 2.2% for all study participants and 1.5% among those without a reported history of COVID-19. Previously reported rates in HCWs ranged from 1.6% in Germany^4^ to 13.7% in New York City.^5^ The significant variability in seroprevalence may be due to a combination of local prevalence rates, PPE use and supply, study protocols, and serology platforms. Unfortunately, there have been no comparative local community prevalence studies performed in San Diego. However, a recent large US survey indicated a seroprevalence rate of 6–24 times than the number of reported infections.^2^ Using this ratio of reported infections to seroprevalence, the 6,315 total cases in San Diego County at the start of our study^6^ with a population of 3.338 million would correspond to a community prevalence estimate of 1.1% to 4.5%. Thus, even among high-risk HCWs, the risk of exposure may not be significantly higher than the general population.

Our findings add to a recent study in Michigan that found no significant differences in seroprevalence between frontline workers and non-frontline workers,^7^ indicating that hospital infection control measures appear to be effective. Additionally, this study found that a known community exposure to COVID-19 was associated with a 4.53 times increased odds of being seropositive, which nearly matches our results. Surprisingly, we found that Hispanic/Latino individuals also had increased odds of being seropositive, independent of other potential risk factors. The reason for this is unclear and will require further investigation for potential confounding factors, such as detailed history of exposures in the community.

Our study has a number of important limitations. First, our study focused only on high-risk HCWs and did not include a comparative group of lower-risk individuals. Second, we did not perform a longitudinal seroconversion analysis to assess effects of infection control measures over time. Third, the responses to our survey were subject to recall bias and errors in self-reporting. Fourth, enrollment into our study was voluntary and subject to self-selection bias since mandated random sampling was impractical. Fifth, due to multiple comparisons of this explorative investigation, we may be underpowered to identify significant associations.

In summary, in this study, a relatively small proportion of HCWs in high-risk care areas were seropositive for SARS-CoV-2. This finding suggests that the appropriate use of PPE is effective in minimizing exposures at the bedside. Additional attention on minimizing COVID-19 exposure beyond the bedside may further help protect the healthcare workforce.
